# Characterization of the disease-causing mechanism of KIF3B mutations from ciliopathy patients

**DOI:** 10.3389/fmolb.2024.1327963

**Published:** 2024-04-11

**Authors:** Jessica M. Adams, Caleb Sawe, Skye Rogers, Jordyn Reid, Ronith Dasari, Martin F. Engelke

**Affiliations:** School of Biological Sciences, Cell Physiology, Illinois State University, Normal, IL, United States

**Keywords:** Kif3B, kinesin-2, ciliogenesis, retina degeneration, ciliopathy, rigor kinesin, structure/function

## Abstract

The heterodimeric kinesin-2 motor (KIF3A/KIF3B with accessory protein KAP3) drives intraflagellar transport, essential for ciliogenesis and ciliary function. Three point mutations in the KIF3B subunit have recently been linked to disease in humans (E250Q and L523P) and Bengal cats (A334T) (Cogné et al., Am. J. Hum. Genet., 2020, 106, 893–904). Patients display retinal atrophy and, in some cases, other ciliopathy phenotypes. However, the molecular mechanism leading to disease is currently unknown. Here, we used *Kif3a* ^*−/−*^ *;Kif3b* ^−/−^ (knockout) 3T3 cells, which cannot make cilia, to characterize these mutations. While reexpression of KIF3B(E250Q) and KIF3B(L523P) did not rescue ciliogenesis, reexpression of wildtype or KIF3B(A334T) restored ciliogenesis to wildtype levels. Fluorescent tagging revealed that the E250Q mutant decorated microtubules and thus is a rigor mutation. The L523P mutation, in the alpha-helical stalk domain, surprisingly did not affect formation of the KIF3A/KIF3B/KAP3 complex but instead impaired motility along microtubules. Lastly, expression of the A334T motor was reduced in comparison to all other motors, and this motor displayed an impaired ability to disperse the Golgi complex when artificially linked to this high-load cargo. In summary, this work uses cell-based assays to elucidate the molecular effects of disease-causing mutations in the KIF3B subunit on the kinesin-2 holoenzyme.

## 1 Introduction

Cilia are microtubule-based projections extending from most cells of the human body. There are two classes of cilia: motile and immotile (primary) cilia. Motile cilia can move extracellular fluid over epithelia, as observed in the lung or brain, or propel cells through fluid, such as sperm cells. Primary cilia function as sensory organelles involved in transducing signals from the environment or other cells. They can also be enriched with specific growth factor or morphogen receptors, making them critically important for normal development and tissue differentiation (Mill et al., 2023). Due to their ubiquity and diverse functions, ciliary defects can lead to diseases with a wide range of symptoms involving multiple systems, generalized with the term ciliopathies (Reiter and Leroux, 2017).

**FIGURE 1:**
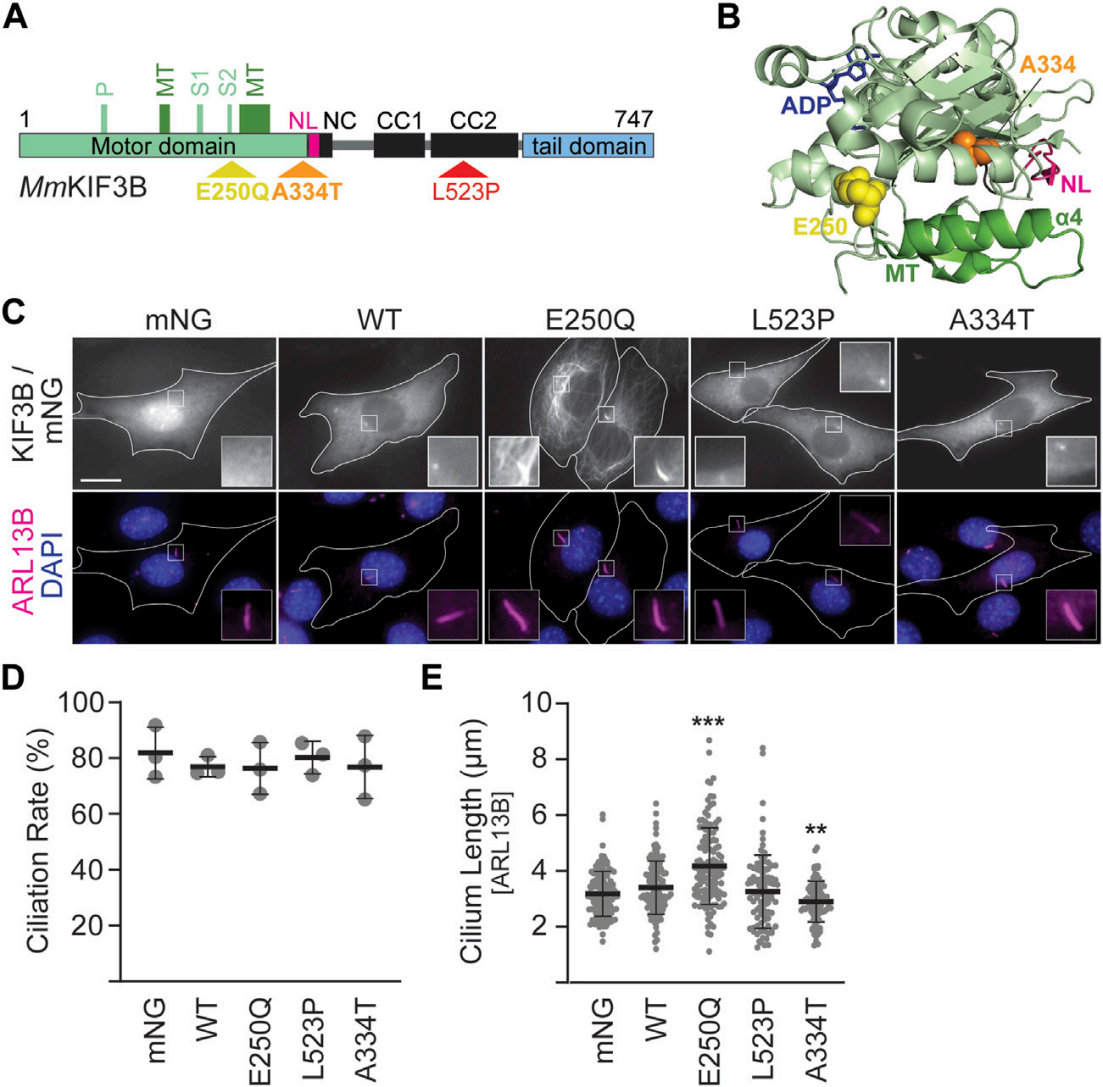
Mutant KIF3B expression in wildtype background has subtle effects on cilia lengths. **(A)** Schematic of the *Mm*KIF3B protein illustrating the location of each disease-causing mutation (E250Q, A334T, and L523P) in standard one letter amino acid abbreviation. The motor domain (light green), neck linker (pink, NL), coiled-coil regions of the stalk domain (black; NC, neck coil; CC1 and 2, coiled-coil 1 and 2), and tail domain (light blue) are indicated. Furthermore, components of the ATP binding motif (P, P-loop; S1/S2, Switch 1 and 2 regions) as well as the microtubule binding interface (dark green, MT) are annotated. Small, black numbers denote amino acid position. **(B)** Crystal structure of *Hs*KIF3B (3B6U) illustrating the location of the residues A334 (orange spheres) and E250 (yellow spheres) that cause disease when mutated. NL: neck linker, pink; MT: microtubule-binding interface, dark green. **(C–E)** Wildtype (WT) 3T3 cells were transfected with wildtype or mutant KIF3B-mNeonGreen (mNG) or soluble mNG. Primary cilium formation was induced by serum starvation for 48 h. **(C)** Representative images of each condition. Cilia were visualized via an antibody against the ciliary membrane marker ARL13B (magenta), and nuclei were visualized with DAPI (blue). Insets show an enlarged region of interest around the cilium in the mNG and ARL13B channel. Scale bar = 15 µm. **(D)** Quantification of the ciliation rate (percentage of transfected cells with a cilium) in each condition. One-way ANOVA revealed no significant difference between groups (F (4,10) = 0.26, *p* = 0.89). **(E)** Quantification of cilium lengths, which was measured in the ARL13B channel. One-way ANOVA revealed a significant difference between groups (F (4,552) = 23.09, *p* < 0.001). **p < 0.01; ***p < 0.001 compared to WT according to Dunnett’s multiple comparison *post hoc* tests. Data are from three independent experiments. N > 115 transfected cells per condition and data are presented as mean ± SD.

KIF3B is a member of the kinesin family (KIF) proteins that forms a heterodimer with KIF3A, which can also dimerize with KIF3C. Both heterodimers can further associate with the accessory protein KAP3. While the KIF3A/KIF3C/KAP3 motor is thought to mainly function in neurons, the KIF3A/KIF3B/KAP3 motor is most prominently known for driving intraflagellar transport (IFT) (Scholey, 2013). IFT, the bidirectional motor-based transport of macromolecular complexes along the ciliary axoneme, is essential to assemble and maintain cilia. In mammals the KIF3A/KIF3B/KAP3 motor is required for anterograde transport, while retrograde transport is powered by dynein-2 (Morthorst et al., 2018). KIF3A and KIF3B are each composed of an N-terminal motor domain that couples ATP hydrolysis to stepwise advancement along microtubules, alpha-helical stalk regions forming heterodimeric coiled-coil segments, thus dimerizing the motor, and unstructured C-terminal tail domains that bind cargo and are involved in autoinhibition (Figure 1A). The accessory protein KAP3 binds to the coiled-coil and/or tail of the KIF3A/KIF3B complex and is suggested to participate in motor regulation and cargo binding (Webb et al., 2020).

Three mutations in KIF3B were recently identified and shown to cause ciliopathies (Cogné et al., 2020). Throughout this article, we will refer to amino acids using the standard one-letter code. E250Q is a *de novo* mutation in one patient who exhibited symptoms involving multiple organ systems, including the retina, skeleton, liver, and heart. L523P was identified as the cause of previously described autosomal-dominant retinitis pigmentosa in an extended Ashkenazi Jewish family (Sullivan et al., 2006). Finally, A334T was found to cause progressive retinal atrophy in a colony of Bengal cats in a recessive fashion.

Here, we delineate the effect of these disease-causing KIF3B mutations on the properties of the kinesin-2 holoenzyme. For this, we expressed fluorescently-tagged mutants in wildtype (WT) and *Kif3a* ^*−/−*^ *;Kif3b* ^*−/−*^ mouse embryonic fibroblasts and delineated their intracellular localization profile as well as their effects on ciliogenesis rate and cilia length. Based on these results and structural information, we then used specific cell-based assays to characterize each mutation further. We identified both autosomal dominant mutations, E250Q and L523P, as mutations that prevent the affected motor from carrying out its natural function to processively advance along microtubules and drive ciliogenesis. In contrast, expression of the A334T motor did not alter ciliogenesis rates but resulted in reduced KIF3B expression levels and impaired the ability of the motor to transport under high load. Understanding how these mutations affect kinesin-2 function will contribute to a better understanding of the anterograde IFT motor in normal physiology and disease.

## 2 Materials and methods

### 2.1 Plasmids

*Mm*KIF3A, *Mm*KIF3A-mNeonGreen (mNG), *Mm*KIF3B-mCherry (mCh), and *Mm*KIF17-mNG have been previously described (Engelke et al., 2019). All primers used to generate constructs are listed in Supplementary Table S1. pmNeonGr-N1 (soluble mNG) was generated by PCR amplifying the mNeonGreen (Shaner et al., 2013) (Allele Biotechnologies) open reading frame (ORF) from *Mm*KIF3A-mNG, adding an AgeI and Kozak consensus sequence on the 5′ end and inserting it into pmCherry-N1 (Clontech Cat# 632523) via AgeI and BsrGI. *Mm*KIF3B-mNG was generated by replacing the mCh of *Mm*KIF3B-mCh with the mNG of *Mm*KIF3A-mNG using BamHI and BsrGI. The mutations E250Q, A334T, and L523P were incorporated into *Mm*KIF3B-mNG via mutagenesis by overlap extension (MOE) PCR (Ho et al., 1989) using BclI and SpeI (E250Q), BclI and PstI (A334T), and PstI and MluI (L523P). myc-mNG-*Mm*KIF3A was generated by synthesizing DNA encoding the myc ORF [(M)EQKLISEEDL], the linker GSGSGRP, mNG, the linker RTGSGSGSGP, and the N-terminal sequence of *Mm*KIF3A, and then inserting it into the *Mm*KIF3A vector via NheI and SalI. DNA sequences encoding the ORF of *Mm*KAP3A (NP_001292572.1) and *Mm*KAP3B (NP_034759) were codon optimized for mouse expression, synthesized, and cloned into pmNeonGr-N1 with NheI and AgeI. Subsequently, the ORF of TagBFP (from *Mm*KIF3B(1–414)-TagBFP, a gift from K. Verhey) was amplified via PCR, adding an AgeI site and short linker (final linker: GPVGS) on the 5′ end and a stop codon and NotI site on the 3′ end, then used to replace the mNG via AgeI and NotI. Myc-mNG-*Mm*KIF17 was generated by first removing the C-terminal mNG from *Mm*KIF17-mNG by introducing a stop codon via PCR amplification of the 3′ end of KIF17 and cloning the fragment back into *Mm*KIF17-mNG via PstI and NotI. Then, the NEBuilder HiFi system was used to insert the N-terminal myc-mNG sequence from myc-mNG-*Mm*KIF3A into the *Mm*KIF17 plasmid that had been digested with NheI and BsiWI. *Mm*KIF3B_L523P-mCh was generated by replacing the mNG of *Mm*KIF3B_L523P-mNG with mCh from *Mm*KIF3B-mCh via BamHI and BsrGI. KIF3A/B^SWAP^ constructs were generated via splice by overlap extension PCR (Horton et al., 1989) from *Mm*KIF3A-mNG and *Mm*KIF3B-mCh and cloned with NheI and XhoI (*Mm*KIF3A (1–359)-3B (355–747)-mCh into *Mm*KIF3B-mCh) or NheI and PstI (*Mm*KIF3B(1–354)-3A (360–701)-mNG into *Mm*KIF3A-mNG). *Mm*KIF3A(1–359)-3B (355–747)_L523P-mCh was generated by cloning the section of KIF3B containing the mutation from *Mm*KIF3B_L523P-mCh into *Mm*KIF3A(1–359)-3B(355–747)-mCh via XhoI and MluI. *Rn*KIF5C(1–559)-mCh-GTS and DMRB-*Rn*KIF5C(9–559)-mCh-GTS were generated by replacing the mCitrine from previously described plasmids (Engelke et al., 2016) with mCherry via AgeI and BsrgI. *Mm*KIF3A(1–359)-3B(355–747)-mCh-GTS was generated by cloning the C-terminal portion of mCherry and the GTS from *Rn*KIF5C(1–559)-mCh-GTS into *Mm*KIF3A (1–359)-3B (355–747)-mCh via SbfI and MfeI. *Mm*KIF3B(1–354)-3A(360–701)_A334T-mNG was generated by MOE PCR using *Mm*KIF3B(1–354)-3A (360–701)-mNG as a template and backbone. All plasmids were confirmed by sequencing.

### 2.2 Cell culture

Wildtype Flp-in 3T3 (Thermo Scientific Cat# R76107), *Kif3a* ^*−/−*^ *;Kif3b* ^*−/−*^ 3T3 (Engelke et al., 2019), and COS-7 (ATCC Cat# CRL-1651) cells were cultured in D-MEM (Corning Cat# 15017CV) supplemented with 10% Hyclone FetalClone III serum (FCS) (Cytiva Cat# SH30109.03) and 4 mM L-glutamine (Alfa Aesar Cat# J6057322) at 37°C and 5% CO_2_.

For ciliation and peripheral accumulation experiments, 3T3 cells were seeded at a density of 1 × 10^5^ cells/well on glass coverslips (Electron Microscopy Sciences Cat# 7222901) inserted into wells of a 12-well plate (Fisher Scientific Cat# FB012928). For Golgi dispersion experiments, COS-7 cells were seeded at a density of 4 × 10^4^ cells/well as above. Approximately 16 h (3T3) or 6 h (COS-7) later, media was switched to starvation media (1% FCS), and 800 ng total plasmid DNA per well was transfected. 48 h (3T3) or 16 h (COS-7) later, cells were fixed and processed for immunofluorescence (see below). All transfections were performed with Lipofectamine 2000 (Thermo Fisher Scientific Cat# 11668019) according to the manufacturer’s protocol.

### 2.3 Immunofluorescence

Cells were fixed with 10% buffered formalin (Fisher Scientific Cat# 23–305510) for 15 min, and immunostaining was performed as previously described (Engelke et al., 2019). Primary antibodies used were: rabbit polyclonal anti-ARL13B (Proteintech Cat# 17711-1-AP, RRID:AB_2060867; 1:1000); mouse monoclonal anti-acetylated tubulin (Sigma-Aldrich Cat# T6793, RRID:AB_477585; 1:10000); rabbit polyclonal anti-GOLGB1 (Thermo Fisher Scientific Cat# PA5-52841, RRID:AB_2642110; 1:1000). Secondary antibodies used were: goat anti-rabbit-AlexaFluor 647 (Thermo Fisher Scientific Cat# A-21244, RRID:AB_2535812; 1:500); goat anti-mouse-AlexaFluor 568 (Thermo Fisher Scientific Cat# A-11031, RRID:AB_144696; 1:500). Nuclei were visualized with DAPI (Biotium Cat# 40043; 1:10,000). Coverslips were mounted using Prolong Gold (Thermo Fisher Scientific Cat# P36930) onto microscope slides (Fisher Scientific Cat# 12–544-7).

### 2.4 Microscopy and analysis

Images were acquired using an inverted, semi-automated BZ-X810 epifluorescence microscope (Keyence) equipped with a Plan Apochromat 40× objective with 0.95 numerical aperture (NA). Exposure times were optimized for each channel and then kept constant across conditions within each experiment, while all other camera settings were identical across the study. Images were analyzed with ImageJ software enhanced by the FIJI package (Schindelin et al., 2012). For ciliation experiments, low-to medium-expressing cells were selected for analysis by measuring average fluorescence intensity in a small circular cytoplasmic ROI in transfected cells. Ciliation rates were calculated as the percentage of transfected cells with a cilium ≥0.5 µm. Cilium lengths were measured manually and have been non-destructively annotated in the dataset published with this study. Peripheral accumulation was assayed by measuring mean fluorescence intensity in a small circular ROI (0.32 µm^2^) at the cell periphery (F_periphery_) and another next to the nucleus (F_center_). The accumulation ratio was obtained by dividing F_periphery_/F_center_. Two such values were obtained per cell and averaged. Analysis for the Golgi dispersion assay was performed on cells expressing similar levels of mNG and mCh across conditions as previously described (Engelke et al., 2016).

### 2.5 Visible immunoprecipitation (VIP) assay

VIP assay was performed as described with modifications (Katoh et al., 2018). COS-7 cells were seeded in a 6-well plate (Corning Cat# 3516) at a density of 3.6–4 × 10^5^ cells/well. Fresh media was applied 5–6 h later, and 1.6 µg total plasmid DNA per well was transfected via Lipofectamine 2000 according to the manufacturer’s protocol. Approximately 18 h later, construct expression levels were checked via an epifluorescence microscope (Keyence), and lysates were collected according to a previously published protocol (Engelke et al., 2016). Lysates were incubated with anti-c-myc agarose bead slurry (Thermo Fisher Scientific Cat# 20168), pre-washed in Lysis Buffer, for 2.5 h at 4°C with gentle rocking. Subsequently, beads were washed twice with Lysis Buffer, resuspended in 25 µL Lysis Buffer, and loaded into flow chambers prepared by attaching clean #1 coverslips (VWR Cat#112614–9) to microscope slides using two layers of double-sided adhesive tape (Tesa Cat# 05338). Beads were immediately imaged using the Keyence epifluorescence microscope and a PlanFluor 10× objective with 0.30 NA using exposure times optimized for each channel and identical camera settings across conditions.

### 2.6 Statistics

Image sets were blinded to the investigator performing the analysis using a custom-programmed ImageJ macro. At least 30 cells per condition were analyzed per experiment, and the experiment was repeated three times. Prism 10.1.1 (GraphPad) was used to graph the data and perform statistical analyses. Data are displayed as mean ± SD and were analyzed by either Student’s unpaired t-test or one-way ANOVA as outlined in the corresponding figure legends. Following a significant F-value, Dunnett’s *post hoc* comparison tests were used to identify groups that were significantly different from the control. Details, including N values and F-values, can be found in the figure legends.

### 2.7 Data availability statement

Plasmids generated for this study are available upon reasonable request. All plasmids encoding a mNeonGreen fusion protein are restricted from distribution through an MTA with Allele Biotechnology. The image sets generated and analyzed for this study can be found in the BioImage Archive (Hartley et al., 2022); https://www.ebi.ac.uk/bioimage-archive/) via the identifier S-BIAD1044.

## 3 Results

### 3.1 Expression of fluorescently-tagged KIF3B mutants reveals defects in motor function

The amino acids E250, L523, and A334 in the generally highly conserved KIF3B motor are identical in *Homo sapiens*, *Felis catus,* and *Mus musculus* (Supplementary Figure S1; Cogné et al., 2020). E250 and A334 are found in the motor domain, whereas L523 is in the coiled-coil region (Figures 1A,B). We introduced each mutation into constructs driving the expression of *Mm*KIF3B fused to the fluorescent protein mNeonGreen (mNG). To assess expression levels of the mutant motors on a population level, we expressed them in *Kif3a* ^*−/−*^ *;Kif3b* ^*−/−*^ 3T3 cells and performed a Western blot on the cytosolic fraction of whole cell lysates using a KIF3B antibody. Although relative expression levels varied across experiments, the KIF3B(A334T) motor consistently displayed lower expression than wildtype (WT) control (Supplementary Figure S2). To control for this population-level expression difference, we selected cells with similar mNG and/or mCherry (mCh) fluorescence intensity, as appropriate, across all conditions for all cell-based assays.

We next expressed the constructs in WT 3T3 cells, stimulated ciliogenesis via serum starvation and visualized cilia by immunostaining against the ciliary membrane marker ARL13B (Figure 1C). Soluble mNG served as a negative control. Quantification of the ciliation rate, defined as the percentage of mNG-expressing cells in which a primary cilium could be detected, showed no difference between cells expressing the WT or mutant motors (Figure 1D). However, cells expressing KIF3B(E250Q) displayed a significant increase in average cilium length, while cells expressing KIF3B(A334T) had a significantly decreased average cilium length compared to cells expressing WT KIF3B. No difference in average cilium length was observed in cells expressing KIF3B(L523P) (Figure 1E).

Fluorescently-tagged kinesins appear as a homogeneous cytoplasmic signal excluded from the nucleus (Figure 1C, WT panel). This distribution is caused by the majority of a specific kinesin population existing in a freely diffusive, autoinhibited state (Hollenbeck, 1989; Cai et al., 2007; Hammond et al., 2010). However, the KIF3B(E250Q) motor exhibited a different distribution pattern resembling microtubule staining. Confocal microscopy confirmed that the KIF3B(E250Q)-mNG fusion protein entirely overlapped with immunostained β-tubulin (Supplementary Figure S3), suggesting that the E250Q mutant is bound to microtubules. As such, KIF3B(E250Q) is a newly identified rigor mutant.

**FIGURE 2:**
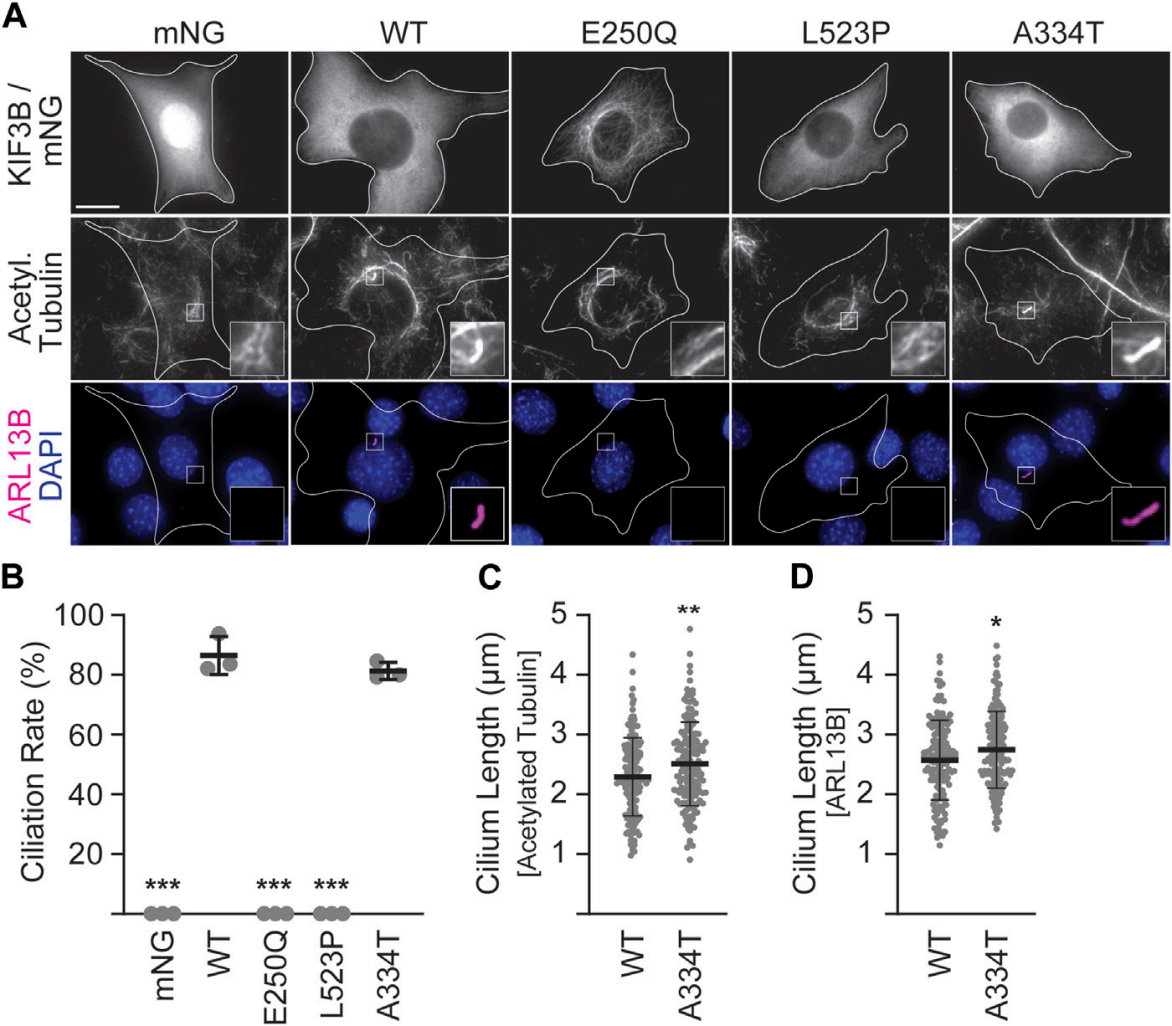
Mutant KIF3B expression in knockout background reveals that E250Q and L523P are loss of function mutations. *Kif3a* ^*−/−*^;*Kif3b* ^*−/−*^ 3T3 cells were transfected with unlabeled wildtype (WT) KIF3A and WT or mutant KIF3B-mNeonGreen (mNG) or soluble mNG. Primary cilium formation was induced by serum starvation for 48 h. **(A)** Representative images of each condition. Cilia were visualized via antibodies against acetylated tubulin (middle panel) and the ciliary membrane marker ARL13B (lower panel, magenta), and nuclei were visualized with DAPI (blue). Insets show an enlarged region of interest of the acetylated-tubulin and ARL13B channels. Scale bar = 15 µm. **(B)** Quantification of the ciliation rate (percentage of transfected cells with a cilium) in each condition. One-way ANOVA revealed a significant difference between groups (F (4,10) = 649.9, *p* < 0.001). ***p < 0.001 compared to WT according to Dunnett’s multiple comparison *post hoc* tests. **(C, D)** Quantification of cilium lengths in the acetylated tubulin and ARL13B channels. Unpaired Student’s t-test revealed a significant difference between groups using both **(C)** acetylated tubulin (t (316) = 2.91; **p = 0.004) and **(D)** ARL13B (t (316) = 2.4, *p = 0.02) as the ciliary marker. Data are from three independent experiments. N > 170 transfected cells per condition and data are presented as mean ± SD.

In the absence of exogenously supplied motors, *Kif3a* ^*−/−*^ *;Kif3b* ^*−/−*^ 3T3 cells cannot make cilia, whereas co-expression of WT KIF3A and WT KIF3B rescues ciliation to WT levels (Figure 2B; Engelke et al., 2019). Thus, to assay the ability of the mutant KIF3B subunits to rescue wildtype motor function, we co-expressed each mutant with WT *Mm*KIF3A in *Kif3a* ^*−/−*^ *;Kif3b* ^*−/−*^ 3T3 cells. We then stimulated ciliogenesis as above, performed immunostaining for two ciliary markers, ARL13B and the acetylated tubulin of the axoneme, and found that both the E250Q and the L523P mutants were unable to produce cilia (Figures 2A,B). The ciliation rate of the A334T mutant was indistinguishable from WT, although the cilia surprisingly measured slightly longer than WT using both the acetylated tubulin as an axonemal and ARL13B as a ciliary membrane marker for measuring (Figures 2C,D).

### 3.2 The KIF3B (L523P) mutation impairs kinesin-2 motility

**FIGURE 3:**
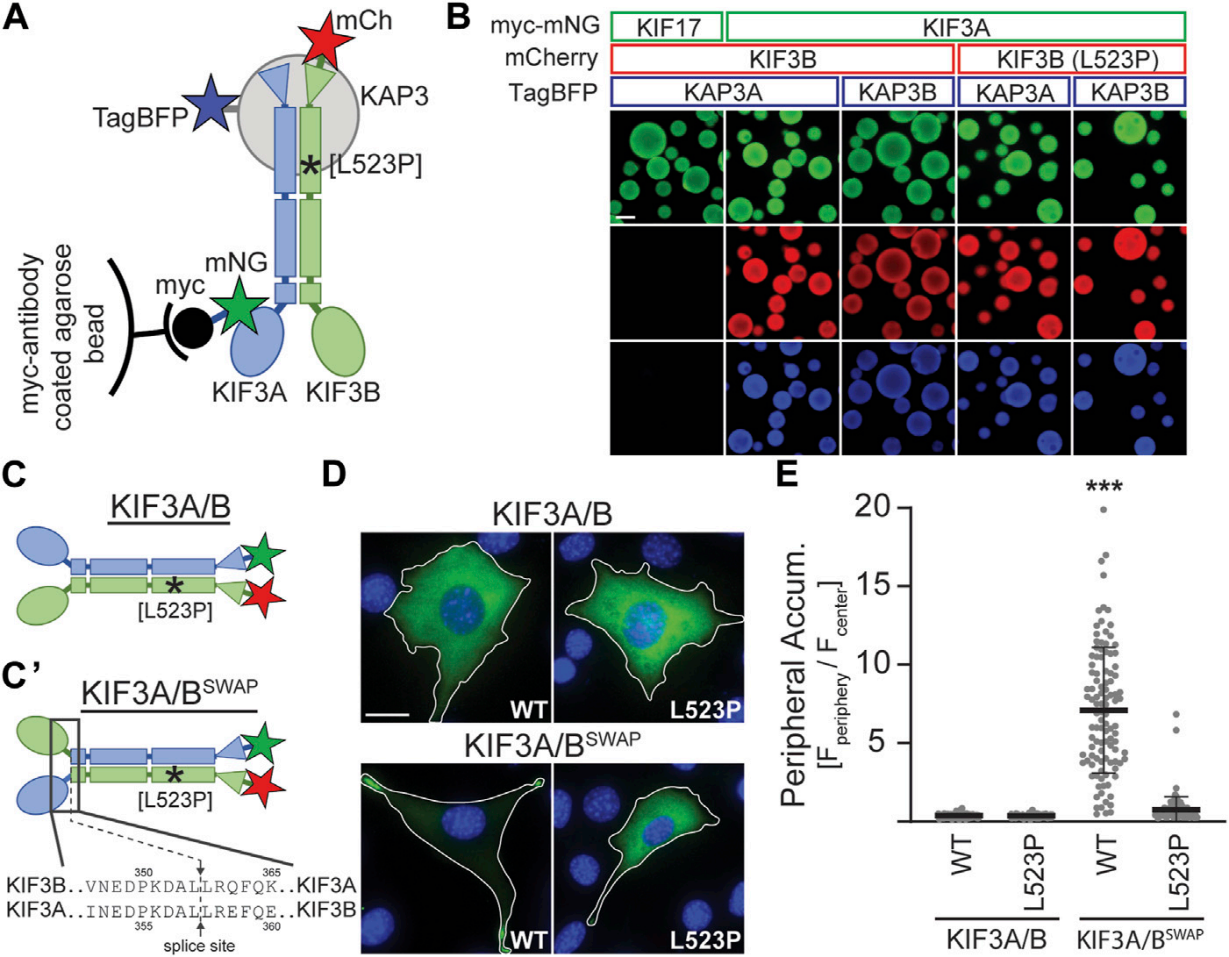
KIF3B mutation L523P does not affect KIF3A/KIF3B/KAP3 complex formation but impairs motor motility. **(A)** Schematic of the Visible Immunoprecipitation (VIP) assay. Whole cell lysates from COS-7 cells transfected with either myc-mNG-KIF17 (negative control) or myc-mNG-KIF3A as well as KIF3B-mCh and KAP3-TagBFP were prepared and incubated with myc-antibody coated agarose beads. Asterisk marks approximate location of the L523P mutation. **(B)** Representative images of beads. Scale bar = 100 µm. **(C)** Schematic illustrating differences between the wildtype KIF3A/B and the constitutively active swap KIF3A/B^SWAP^ kinesin-2 motors. The KIF3A/B^SWAP^ motor **(C’)** results from co-expressing chimeric subunits in which the motor domain and neck linker of KIF3A and KIF3B have been fused to the stalk and tail domain of KIF3B and KIF3A, respectively. The splice sites are indicated. Asterisk marks approximate location of L523P mutation. **(D)** Representative images of *Kif3a* ^*−/−*^ *;Kif3b* ^*−/−*^ 3T3 cells transfected with kinesin-2 constructs described in panels C and C'. mNG (green) and DAPI (blue) are shown; for mCherry (mCh) images, see Supplementary Figure S4. Top, KIF3A/B was expressed via co-transfection of plasmids encoding KIF3A-mNG and either WT or L523P KIF3B-mCh. Bottom, KIF3A/B^SWAP^ was expressed via co-transfection of plasmids encoding the chimeric subunits with either the WT or L523P KIF3B stalk. Inset label describes the KIF3B genotype. Scale bar = 15 µm. **(E)** Quantification of peripheral accumulation (ratio of mNG fluorescence of a peripheral [F_periphery_] divided by a central [F_center_] region of interest) in transfected cells in each condition. One-way ANOVA revealed a significant difference between groups (F (3,510) = 352.9, *p* < 0.001). ***p < 0.001 compared to WT KIF3A/B according to Dunnett’s multiple comparison *post hoc* tests. Data are from three independent experiments. N > 100 transfected cells per condition and data are presented as mean ± SD.

Based on the location of the L523P mutation in the coiled-coil region of KIF3B (Figure 1A) and the known effect of proline (P) as an α-helix breaker, we tested the hypothesis that the mutation disrupts dimerization of the KIF3B subunit with KIF3A and/or binding of the accessory protein KAP3 to the heterodimer. We employed the Visible Immunoprecipitation (VIP) assay (Katoh et al., 2018) to test the interaction between these three subunits. We prepared spin-clarified cell lysates from COS-7 cells co-expressing myc-mNG-*Mm*KIF3A with WT or mutant *Mm*KIF3B-mCh and TagBFP fused to one of the two splice variants of KAP3 (*Mm*KAP3A and *Mm*KAP3B). Next, we incubated the lysates with anti-myc agarose beads and visualized the fluorescent signal on the beads via epifluorescence microscopy (Figure 3A). As a negative control, we replaced the myc-mNG-*Mm*KIF3A with myc-mNG-*Mm*KIF17, a closely related motor that does not bind to KIF3B or KAP3. Indeed, we found that myc-mNG-*Mm*KIF17 was visible on the beads but did not show any interaction with KIF3B or KAP3A. In contrast, both KIF3B(WT) and KIF3B(L523P) showed strong interaction with KIF3A and both isoforms of KAP3 (Figure 3B). These results suggest that the L523P mutation does not affect the formation of the heterotrimeric KIF3A/KIF3B/KAP3 complex.

Since the L523P mutant could not rescue ciliogenesis, we aimed to test if a motor incorporating the L523P mutation can move processively along microtubules. Motor motility can be tested in cell-based assays by monitoring the accumulation of constitutively active kinesins in the cell periphery (Hammond et al., 2009). Based on a report from the kinesin-2 homolog in *C. elegans*, KLP11/KLP20 (Brunnbauer et al., 2010), we engineered a constitutively active kinesin-2 motor by fusing the KIF3A motor domain to the KIF3B stalk and tail domain and *vice versa*. Co-expression of these chimeric subunits results in a kinesin-2 motor in which the motor domains have been swapped (KIF3A/B^SWAP^, Figure 3C). When expressed in *Kif3a* ^*−/−*^ *;Kif3b* ^*−/−*^ cells, the KIF3A/B^SWAP^ motor no longer exhibited the homogeneous cytoplasmic expression of the autoinhibited WT motor but accumulated in the periphery of the cell, characteristic of a constitutively active motor (Figure 3D). However, introducing the L523P mutation into the KIF3B stalk domain abrogated this strong peripheral accumulation of the KIF3A/B^SWAP^ motor (Figures 3D, E; Supplementary Figure S4). These data demonstrate that the L523P mutant motor cannot be activated to walk along the microtubules and provide a rationale for its inability to drive ciliogenesis.

### 3.3 The KIF3B (A334T) mutation reduces motor expression and impairs its ability to disperse cargo

**FIGURE 4:**
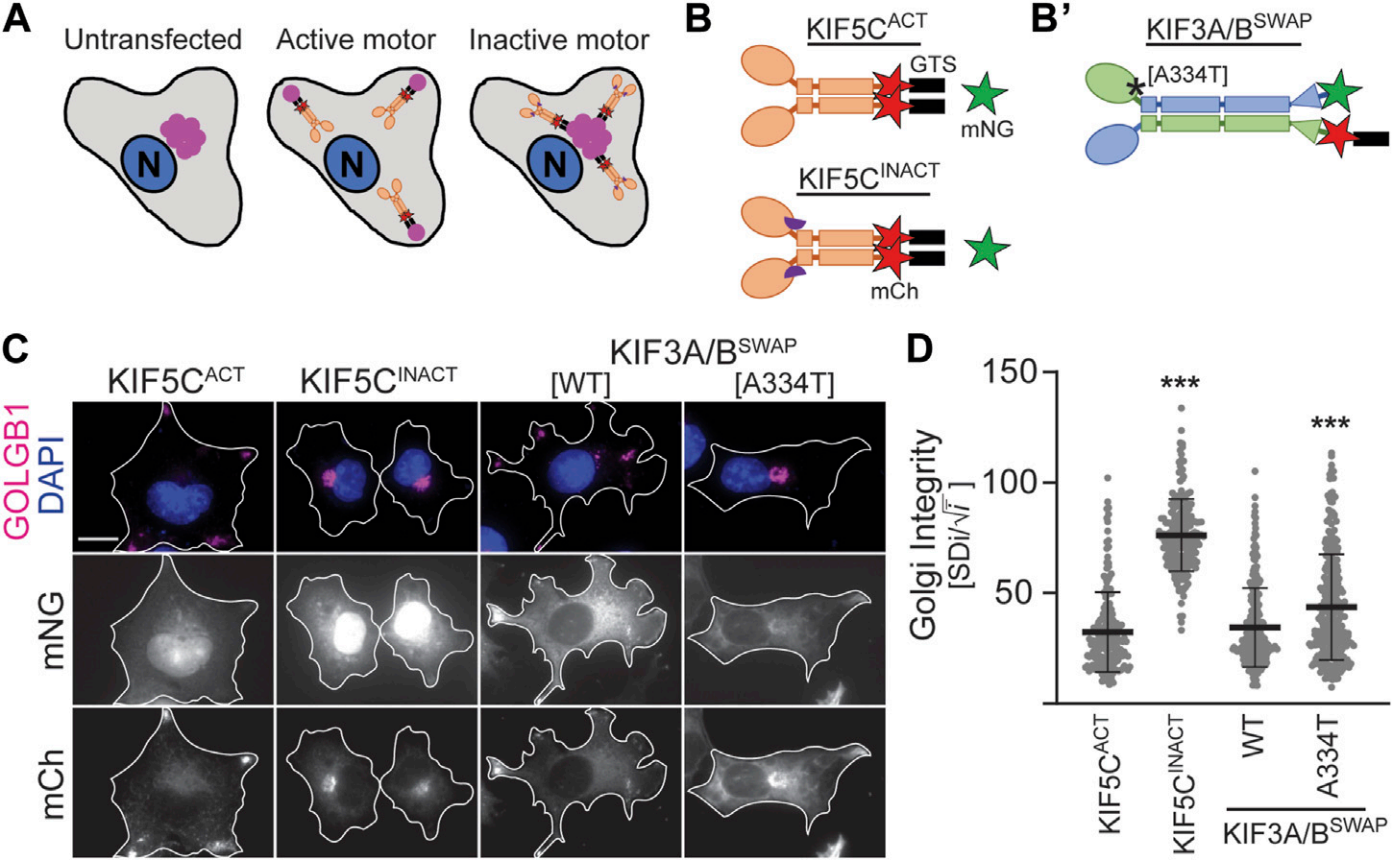
KIF3B mutation A334T limits the motor’s ability to transport artificial high-load cargo. **(A, B)** Schematic of the Golgi dispersion assay and motor constructs used. **(A)** In untransfected cells, the Golgi apparatus (pink) is compact and located in nuclear proximity (left). When transfected cells express constitutively active motors (ochre) fused to a Golgi targeting sequence (GTS, black), the motors disperse the Golgi to the periphery (center). Inactive motors (ochre) fused to a GTS bind to the Golgi but are unable to translocate it from its perinuclear localization (right). N: nucleus. **(B)** A GTS and mCherry (mCh, red) were fused to the C-terminus of either the constitutively active KIF5C^ACT^ [*Rn*KIF5C(1–559)] or inactive KIF5C^INACT^ [DmrB-*Rn*KIF5C(9–559)] kinesin-1 motor. The inactive construct is N-terminally fused to a DmrB domain (purple semicircle), which is not part of the Golgi dispersion assay. **(B’)** A GTS was fused to the C-terminus of the mCh in the KIF3A/B^SWAP^ construct. Asterisk marks approximate location of A334T mutation. **(C)** Representative images of COS-7 cells transfected with either soluble mNG and active or inactive KIF5C-mCh-GTS (left panels) or KIF3A/B^SWAP^-GTS with or without the A334T mutation in the KIF3B motor domain (right panels). The Golgi apparatus was visualized via an antibody against GOLGB1 (magenta), and nuclei were visualized with DAPI (blue). Scale bar = 15 µm. **(D)** Quantification of Golgi dispersion as described in the methods section. Data are presented as Golgi integrity in which a high value reflects tightly packed perinuclear Golgi and a low value reflects a dispersed Golgi. One-way ANOVA revealed a significant difference between groups (F (3,962) = 224.7, *p* < 0.001). ***p < 0.001 compared to KIF5C^ACT^ according to Dunnett’s multiple comparison *post hoc* tests. Data are from three independent experiments. N ≥ 195 transfected cells per condition and data are presented as mean ± SD.

Despite lower expression levels (Supplementary Figure S2), KIF3B(A334T) motors rescued ciliogenesis in *Kif3a* ^*−/−*^ *;Kif3b* ^*−/−*^ cells to the same extent as WT motors and only showed slight differences in cilia length (Figures 2B–D). Based on the position of the mutation in proximity to the force-generating neck linker (Figures 1A, B), we hypothesized that A334T may affect the motor’s ability to generate force. We used the Golgi dispersion assay (Engelke et al., 2016) to test this hypothesis. In undisturbed cells, the activity of Golgi membrane-associated cytoplasmic dynein causes the characteristic compact and perinuclear appearance of the Golgi apparatus. For the Golgi dispersion assay, constitutively active kinesin motors are fused to a Golgi targeting sequence, and their ability to disperse the Golgi apparatus against dynein resistance is measured. Strong and active kinesins succeed in dispersing the Golgi apparatus from its compact and perinuclear location to the periphery of the cell. In contrast, weak or inactive kinesins cannot translocate the Golgi (Figure 4A). As controls, we employed previously published constitutively active KIF5C^ACT^ [*Rn*KIF5C(1–559)] and inactive KIF5C^INACT^ [DmrB-*Rn*KIF5C(9–559)] motors (Figure 4B; Engelke et al., 2016). We found that the constitutively active KIF3A/B^SWAP^ motor (Figure 4B’) could disperse the Golgi to the same extent as KIF5C^ACT^. However, introducing the A334T mutation into KIF3A/B^SWAP^ significantly impaired Golgi dispersion (Figures 4C,D).

## 4 Discussion

Two mutations in human KIF3B, causing disease in an autosomal dominant fashion, were recently identified. A KIF3B mutation recessively causing retinal atrophy in Bengal cats was also reported (Cogné et al., 2020). Using a zebrafish model, the authors of the study linked the observed disease phenotypes to the KIF3B mutations, but the mechanism by which the mutations impair motor function remained elusive. Here, we utilized *Kif3a* ^*−/−*^ *; Kif3b* ^*−/−*^ mouse embryonic fibroblasts and cell-based assays targeting different aspects of motor protein physiology to characterize the specific effect these mutations exert on the kinesin-2 holoenzyme. We found that both the E250Q and L523P mutants were unable to rescue ciliogenesis in *Kif3a* ^*−/−*^ *; Kif3b* ^*−/−*^ knockout cells, suggesting loss of function, whereas the A334T mutant displayed reduced expression and led to a subtle change of cilium length in cultured fibroblasts. The mutations we characterize likely affect the speed and/or efficiency of IFT, as discussed below. While it has been elegantly shown that IFT speed directly affects cilia length (Li et al., 2020), further analysis would be needed to uncover whether the cilium length differences we observe here contribute to pathology.

### 4.1 E250Q is a rigor mutation

Rigor mutations disrupt the chemical-kinetic cycle of the kinesin motor domain, causing it to constantly assume a conformation with high microtubule affinity. This abolishes motor motility and instead leads to a characteristic decoration of the microtubules by the motor (Nakata and Hirokawa, 1995). We observed that the KIF3B(E250Q)-mNG fusion protein decorated microtubules, suggesting that it is a rigor motor. E250 is a highly conserved residue in the kinesin superfamily (Supplementary Figure S5; Cogné et al., 2020). It is located at the C-terminal end of the Switch II motif of the catalytic core, which, together with the P-loop and Switch I, forms an ATP γ-phosphate sensing site considered a main contributor to kinesin motility (Hirokawa et al., 2009). The homologous residue in the human kinesin-1 KIF5B, E236, has been shown to form several nucleotide state-dependent interactions with other residues within the motor domain (Shang et al., 2014; Cao et al., 2017). An engineered kinesin-1 E236A mutant motor was found to be a rigor kinesin since it could not move microtubules in a microtubule gliding assay and was defective in ATP hydrolysis (Rice et al., 1999). Interestingly, several patients with mutations in homologous residues in KIF1A (E253K) and KIF5C (E237K and E237V) have been identified (Esmaeeli Nieh et al., 2015; Lee et al., 2015; Michels et al., 2017), and the E237V mutant was confirmed to be deficient in ATP hydrolysis (Poirier et al., 2013). Based on our results and data from these other mutants, we surmise that the KIF3B E250Q is deficient in ATP hydrolysis, causing it to be a rigor mutant.

### 4.2 The L523P mutation impairs kinesin-2 motility along microtubules

L523 lies in an alpha-helical region of KIF3B that forms a coiled-coil domain with KIF3A that mediates heterodimerization. However, we did not find that the L523P mutation affected the dimerization of KIF3B with KIF3A or the binding of the accessory protein KAP3. This finding is in agreement with a recent study in the *D. melanogaster* KIF3A homolog, KLP64D, in which the authors found that a E551K mutation did not disrupt the formation of the kinesin-2 heterotrimer (Ahmed et al., 2019). Rather, this study suggested that the E551K mutation strengthened the interaction between the alpha helices in the coiled-coil domain, reducing its dynamicity (Ahmed et al., 2020). This could influence neck coil stability and thereby reduce the processive motility of the motor. However, our observation that KIF3B(L523P) motor did not accumulate at the cell’s periphery when autoinhibition was released was a much more dramatic effect than the modest reduction in kinesin-1 run length when the two helices forming the neck coil were cross-linked (Tomishige and Vale, 2000). We currently do not know how the L523P mutation affects the stability of the coiled-coil domain and how changed stability of the neck coil affects motor motility. It is conceivable this parameter affects kinesin-2 differently than kinesin-1, and future work will focus on the specific nature of this defect.

### 4.3 The disease-causing effects of autosomal dominant KIF3B mutations

While homozygous *Kif3b* knockout is embryonic lethal in mice, *Kif3b* ^*+/−*^ mice are healthy without obvious abnormalities, including no increased photoreceptor cell death (Nonaka et al., 1998; Jimeno et al., 2006). It is thus deemed unlikely that loss of function mutations will cause disease via haploinsufficiency. All three KIF3B mutations investigated here (E250Q, A334T, and L523P) were reported to cause retinal degeneration (Cogné et al., 2020), suggesting that photoreceptors are especially sensitive to subtle changes in IFT. This interpretation aligns with the observation that IFT transport capacity is much higher in photoreceptors than in other cilia due to the high amount of required opsin transport (Williams, 2002; Crouse et al., 2014).

We found that co-expression of WT KIF3A with KIF3B(E250Q) or KIF3B(L523P) could not rescue ciliogenesis in *Kif3a* ^*−/−*^ *; Kif3b* ^*−/−*^ cells, and these mutations can thus be classified as loss of function mutations. Even though patients possessed these mutations in heterozygosity, the severity of the observed phenotype was stronger in the E250Q patient. This mutation causes the motor to decorate microtubules, including the ciliary axoneme (Figure 1; Supplementary Figure S3). KIF3B(E250Q) might thereby exert a dominant negative effect by disturbing microtubule dynamics and constituting road blocks for microtubule-dependent transport events (Schneider et al., 2015; Hoeprich et al., 2017; Peet et al., 2018). On the other hand, KIF3B(L523P) motors localize to the base of the cilium (Figure 1C) and potentially bind to IFT trains without contributing to transport, possibly resulting in a dominant negative reduction in the efficiency of IFT transport (Milic et al., 2017). The penetrance of these dominant negative effects will strongly correlate with the expression level of the mutant subunit and might be partly mitigated by cellular mechanisms downregulating the expression of mutant proteins.

### 4.4 The A334T mutation reduce kinesin-2 expression and likely force output

The A334 residue in the motor domain is well-conserved in the kinesin superfamily (Supplementary Figure S5). It is located toward the C-terminal end of alpha helix 6 (α6), where it leads into the neck linker, the mechanical element crucial for motor motility (Rice et al., 1999; Hwang et al., 2008). Both ends of α6 are part of the microtubule-binding interface of the motor domain (Gigant et al., 2013). By acquiring an extra turn when the motor domain transitions from the ADP•Pi to ADP-bound form (Nitta et al., 2008), α6 participates in regulating the microtubule affinity within the chemical cycle. Based on its location on α6 (Figure 1A), we do not believe it likely that the A334T mutation directly alters the microtubule-binding interface. However, since A is a much more common residue in alpha helices than T (Fujiwara et al., 2012), the A334T mutation may impair the introduction of the extra helix turn during phosphate (Pi) release, thereby potentially inhibiting this step in the chemomechanical cycle. Kinetical inhibition of rear head release could conceivably slow the motor, reduce force output, or both.

Recent work in the kinesin-1 KIF5C has uncovered a critical role for neck linker docking in generating force in KIF5C (Hwang et al., 2008; Budaitis et al., 2019), a mechanism that is likely conserved among kinesins (Budaitis et al., 2021). Mutations that disrupt individual interactions between the neck linker and the core motor domain affect motor properties. While motor speed is enhanced, the ability of the motor to transport against load is impaired (Budaitis et al., 2019). The introduction of A334T potentially displaces the C-terminal end of α6 slightly, thereby mispositioning the neck linker so that it cannot form all cognate contacts during docking, causing a force reduction of the power stroke. Both impairments of the chemomechanical cycle or the power stroke are compatible with our observation that introducing the A334T mutation modestly but significantly impaired Golgi dispersal compared to the wildtype motor.

Western blotting revealed overall reduced expression for the A334T motor. To exclude that reduced expression affects the results of the Golgi dispersion assay, we only analyzed cells with similar expression across conditions. Thus, we find it likely that both the reduced expression, perhaps reflecting protein instability, and the impairment in high-load cargo dispersal, which may reflect reduced force output, could impact motor function. The resulting subtle motor impairment likely does not affect IFT in most cilia but renders the motor unable to fully meet the increased IFT demand in the connecting cilia of photoreceptors. This might lead to a gradual build-up of opsin in the inner segment of photoreceptors, eventually leading to the demise of those cells (Crouse et al., 2014).

In summary, we have utilized cell-based assays to test if the disease-causing KIF3B mutations disrupt specific motor properties. The mechanism by which these defects lead to the observed patient phenotypes awaits further characterization. Eventually, this characterization will not only be essential in understanding the basic function of the kinesin-2 motor in health and disease but will also be key to developing strategies to alleviate human disease.

### 4.5 Limitations of the study

We performed functional assays in which motors were expressed in their native cellular environment, which can provide insights that cannot be gleaned from *in vitro* experiments (see, e.g., Chai et al., 2024). However, further data from *in vitro* experiments, which can determine important motor parameters such as run length and velocity, would allow more informed conclusions to be drawn from our data. Additionally, this study used mouse cell lines in conjunction with mouse kinesin-2 motors to model effects of mutations that cause disease in humans and cats. Mouse, cat and human KIF3B share 98% amino acid identity (Supplementary Figure S1), and we have found that ectopic expression of WT human KIF3A/KIF3B constructs rescue ciliogenesis completely in mouse *Kif3a* ^*−/−*^ *; Kif3b* ^*−/−*^ cells (unpublished observation). Furthermore, human skin fibroblasts derived from the E250Q patient exhibited significantly elongated cilia (Cogné et al., 2020), identical to our finding when we expressed the KIF3B(E250Q) motor in WT cells (Figure 1E). Therefore, we find it likely that our results are applicable to human and cat KIF3B, but cannot rule out the possibility that the mutations could lead to different phenotypes in different species.

## Data Availability

The raw data supporting the conclusion of this article will be made available by the authors, without undue reservation.
