# Supplementary material for: Characterization of the disease-causing mechanism of KIF3B mutations from ciliopathy patients
Source: Front Mol Biosci. 2024 Apr 11;11:1327963. doi: 10.3389/fmolb.2024.1327963 (PMC11043552; doi:10.3389/fmolb.2024.1327963)
Supplement: Supplementary file 1 [file DataSheet1.docx]

Supplementary Material

# Supplementary Figures and Tables

## Supplementary Figures


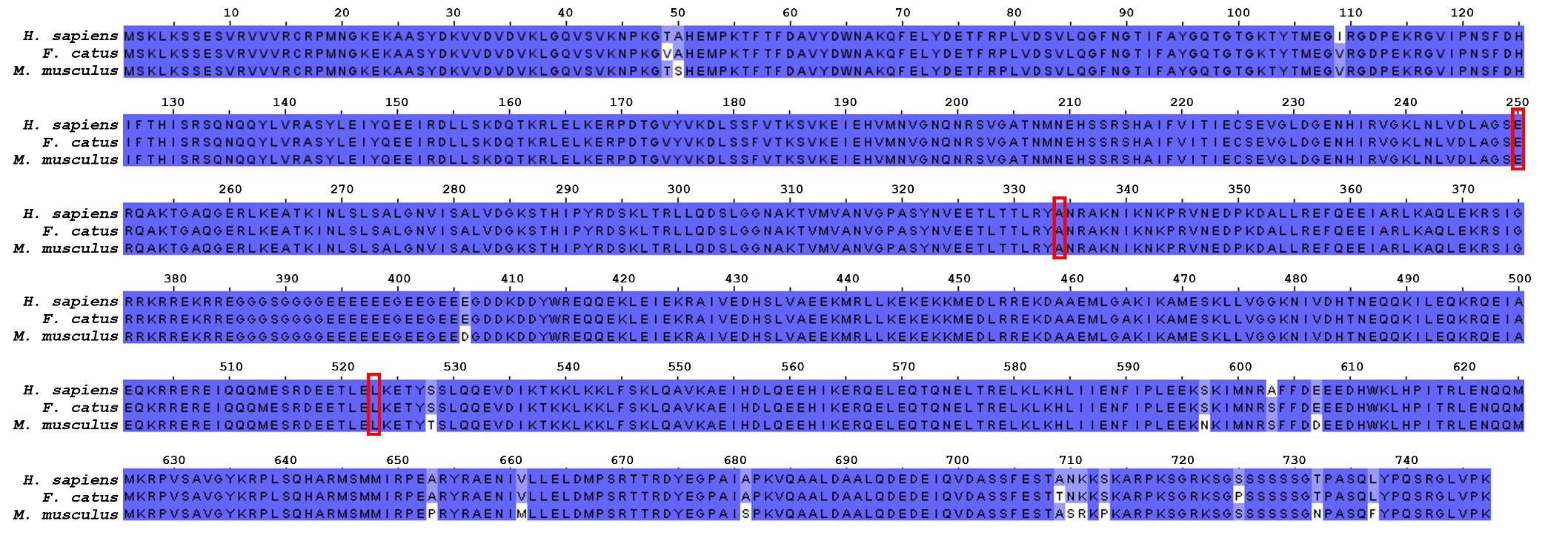


**Supplementary Figure S1.** **KIF3B shows very high sequence conservation across species studied.** Multiple sequence alignment of KIF3B from *Homo sapiens*, *Felis catus,* and *Mus musculus*. Colors indicate percent identity. Residues examined in this study are boxed in red.


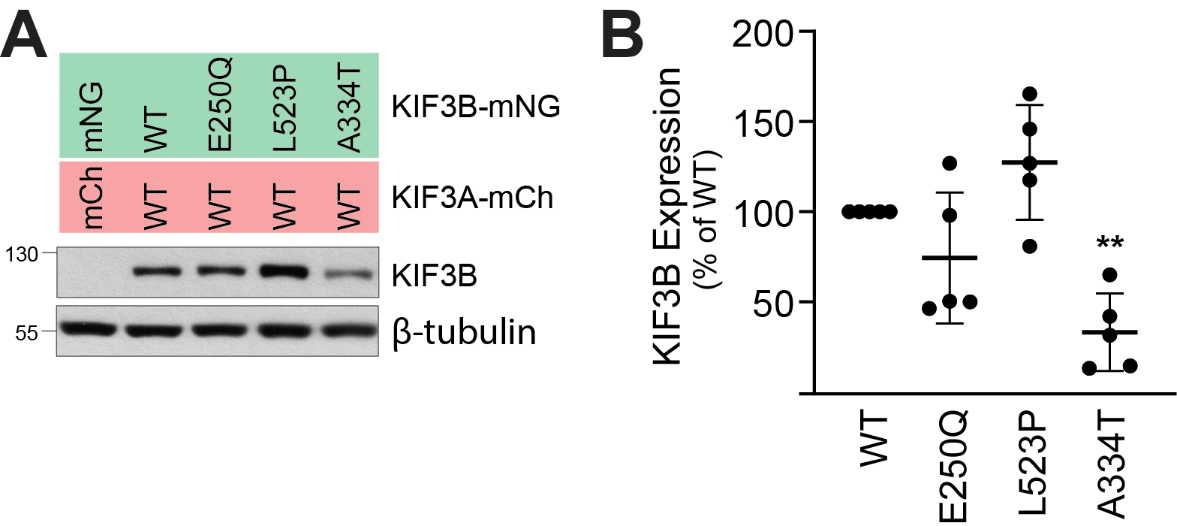


**Supplementary Figure S2.** **KIF3B mutation A334T affects protein expression levels.** *Kif3a^-/-^Kif3b^-/-^* 3T3 cells were transfected with soluble mNG and mCh or wildtype (WT) *Mm*KIF3A-mCh and either WT, E250Q, L523P or A334T *Mm*KIF3B-mNG. Subsequently, crude cellular lysates were collected and analyzed via Western blot. **(A)** Representative image of a blot showing KIF3B-mNG (top) and β-tubulin (bottom) bands. **(B)** Quantification of the band intensity in each condition. KIF3B values were normalized to WT within each experiment. One-way ANOVA revealed a significant difference between groups (F(3,16)=11.5, p<0.001). **p<0.01 compared to WT according to Dunnett’s multiple comparisons post hoc test. Data are from 5 independent experiments and are presented as mean ± SD.

**
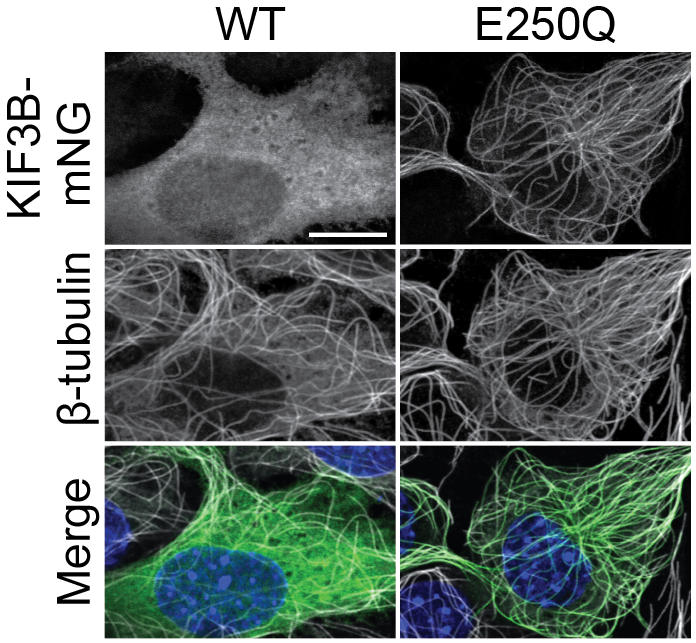
**

**Supplementary Figure S3.** **KIF3B mutation E250Q is a novel rigor mutation.** *Kif3a^-/-^Kif3b^-/-^* 3T3 cells were transfected with unlabeled wildtype (WT) KIF3A and WT (left) or E250Q (right) KIF3B-mNeonGreen (mNG) (top). Subsequently, cells were immunostained with an anti-β-tubulin antibody (middle), and images were acquired on an inverted confocal microscope. In the merged image (bottom), mNG was pseudocolored in green, and nuclei were visualized with DAPI (blue). Scale bar = 10 µm.


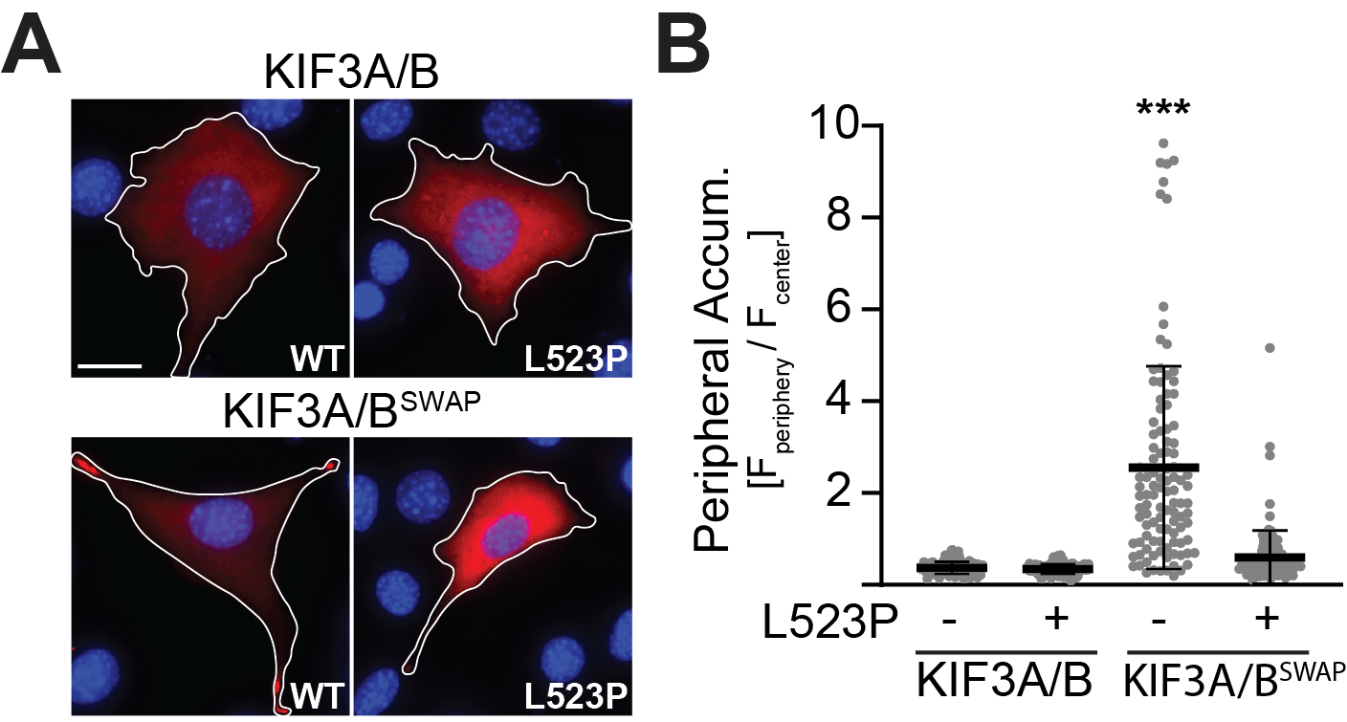


**Supplementary Figure S4. KIF3B mutation L523P impairs motor motility.** These data accompany Figure 3 D and E. **(A)** Representative images of *Kif3a^-/-^Kif3b^-/-^* 3T3 cells transfected with WT and swap motor kinesin-2 constructs described in Figure 3B. mCh (red) and DAPI (blue) from the same images shown in Figure 3D. Top, KIF3A/B was expressed via co-transfection of plasmids encoding KIF3A-mNG and either WT or L523P KIF3B-mCh. Bottom, KIF3A/B^SWAP^ was expressed via co-transfection of plasmids encoding the chimeric subunits with either the WT or L523P KIF3B stalk. The inset label describes the KIF3B genotype. Scale bar = 15 µm. **(B)** Quantification of peripheral accumulation ratio of mCh fluorescence in transfected cells in each condition. One-way ANOVA revealed a significant difference between groups (F(3,510)=115.4, p<0.001). ***p<0.001 compared to WT according to Dunnett’s multiple comparison post hoc tests. Data are from three independent experiments. N>100 transfected cells per condition, and data are presented as mean ± SD.

**
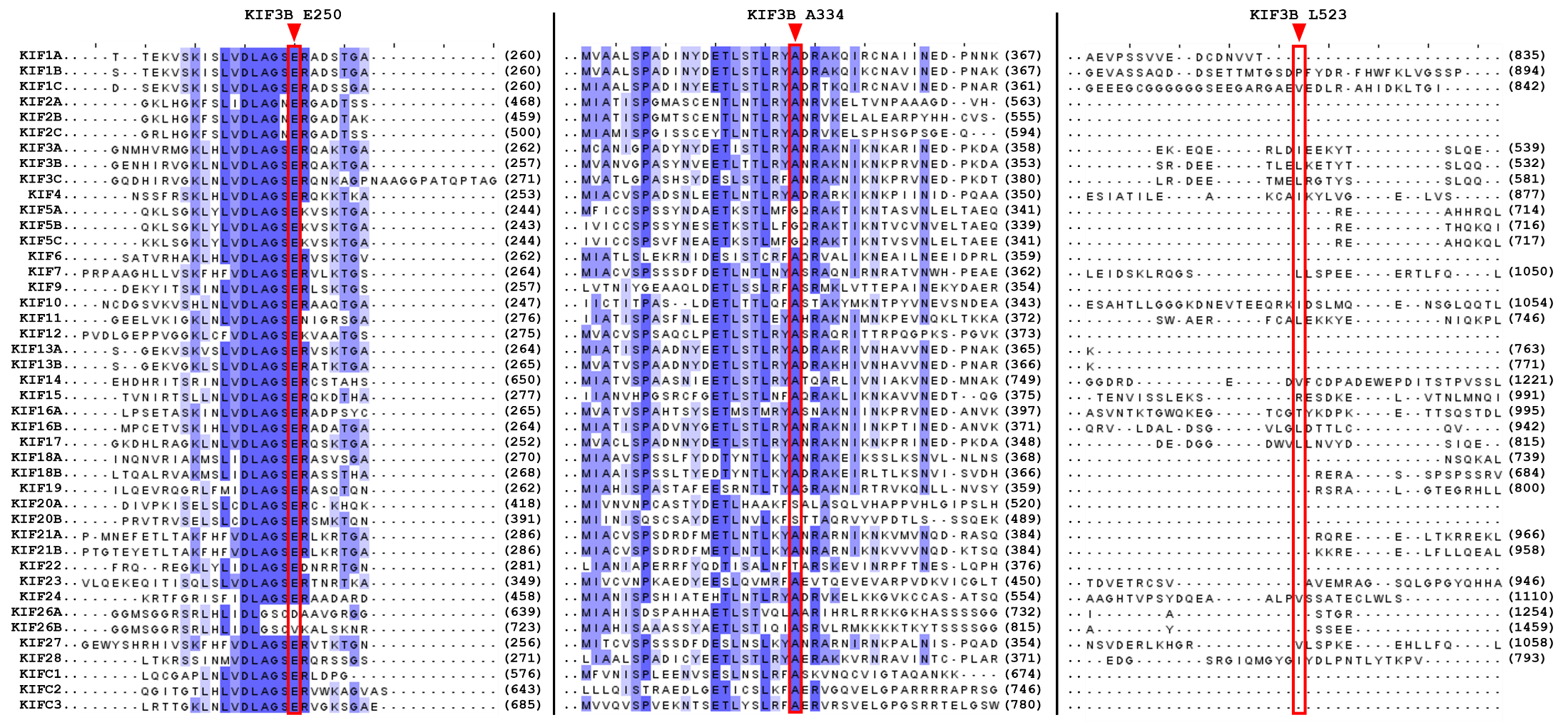
**

**Supplementary Figure S5. Motor domain residues E250 and A334 are highly conserved across mouse kinesins, whereas stalk domain residue L523 is not.** Multiple sequence alignment of 43 mouse kinesins where color indicates percent identity. Residues examined in this study are boxed in red. The number in parentheses on the right corresponds to the position of the last amino acid on that line for that portion of the alignment shown.

## Supplementary Tables

| pmNeonGr-N1 | F | 5’-ttatatACCGGTCGCCACCATGGTGAGCAAGGGCG-3’ |
| --- | --- | --- |
|  | R | 5’-TTACTTGTACAGCTCGTCCATGC-3’ |
| MmKIF3B_E250Q-mNG | F | 5’-TTCAGACCCCTGGTGGACT-3’ |
|  | R | 5’-GCCTGTCTCTgAGATCCGGCCAGGTCCA -3’ |
|  | F | 5’-GCCGGATCTcAGAGACAGGCTAAAACAGGCG-3’ |
|  | R | 5’-TCGCCTTCCTCTTCTTCTTCCT-3’ |
| MmKIF3B_A334T-mNG | F | 5’-CAACAGCTTTGATCACATCTTCACCC-3’ |
|  | R | 5’-GGCTCTGTTGGtGTATCTCAGTGTGGTCAGTGTCTCTT-3’ |
|  | F | 5’-CACACTGAGATACaCCAACAGAGCCAAGAACATCAAGAAC-3’ |
|  | R | 5’-CTTCTGCTCGGCGATTTCTTGC-3’ |
| MmKIF3B_L523P-mNG | F | 5’-GGACGACAAGGACGACTATTGGA-3’ |
|  | R | 5’-TGTATGTCTCTTTGgGTTCGAGGGTTTCCTCGTCG-3’ |
|  | F | 5’-AACCCTCGAACcCAAAGAGACATACACCAGCCTCC-3’ |
|  | R | 5’-GGCCGGATCATCATGCTCATTC-3’ |
| MmKAP3A/B-TagBFP | F | 5’- gcgtatACCGGTTGGATCTATGAGCGAGCTGATTAAGGAG  AACATG-3’ |
|  | R | 5’- gcgtatGCGGCCGCTttaATTAAGCTTGTGCCCCAGTTTGC-3’ |
| MmKIF17 | F | 5’-CAACTACTCCAACCTTGAGAAGATCCG-3’ |
|  | R | 5’-tattatGCGGCCGCtcacctagttaCAGGGGCTCTCCGCC-3’ |
| Myc-mNG-MmKIF17 | F | 5’-ttagtgaaccgtcagatccgGCTAGCACCGGTGCCACCATG |
|  | R | 5’-cttgttcataggccggcagcgtaccaccaccttcacagattcagaggc  CATGGGCCCGCTGCCGCT-3’ |
| MmKIF3A(1-359)-3B(355-747)-mCh | F | 5’-CAAATGGGCGGTAGGCGTGTA-3’ |
|  | R | 5’-TTGGAACTCTCTCAGCAGAGCATCCTTGGGGTCCTCGTT-3’ |
|  | F | 5’-CAAGGATGCTCTGCTGAGAGAGTTCCAAGAAGAGATCGCCA-3’ |
|  | R | 5’-GGCCTTGGAGCCGTACAT-3’ |
| MmKIF3B(1-354)-3A(360-701)-mNG | F | 5’-CAAATGGGCGGTAGGCGTGTA-3’ |
|  | R | 5’-CTGGAACTGTCTCAGCAGAGCATCCTTAGGGTCCTCGTTCAC-3’ |
|  | F | 5’-CCTAAGGATGCTCTGCTGAGACAGTTCCAGAAAGAGATCG  AGGAA-3’ |
|  | R | 5’-TAACTCCTCATAACCATCATTTG-3’ |
| MmKIF3B(1-354)-3A(360-701)_  A334T-mNG | F | 5’-CAACAGCTTTGATCACATCTTCACCC-3’ |
|  | R | 5’-GGCTCTGTTGGtGTATCTCAGTGTGGTCAGTGTCTCTT-3’ |
|  | F | 5’-CACACTGAGATACaCCAACAGAGCCAAGAACATCAAGAAC-3’ |
|  | R | 5’-AGCATCTGGAGTCTCAGCTCG-3’ |

**Supplementary Table S1.** Primers used for cloning.

## Supplementary Methods

**1.3.1 Protein Alignment**

Protein sequences of KIF3B from *Homo sapiens* (O15066), *Felis catus* (M3X169) and *Mus musculus* (Q61771) were obtained from the UniProtKB/Swiss-Prot database (The UniProt Consortium, 2023). All available protein sequences for kinesins from *Mus musculus* were also obtained: KIF1A (P33173), KIF1B (Q60575), KIF1C (O35071), KIF2A (P28740), KIF2B (Q8C0N1), KIF2C (Q922S8), KIF3A (P28741), KIF3C (O35066), KIF4 (P33174), KIF5A (P33175), KIF5B (Q61768), KIF5C: (P28738), KIF7 (B7ZNG0), KIF9 (Q9WV04), KIF10 (CENPE, Q6RT24), KIF11 (Q6P9P6), KIF12 (Q9D2Z8), KIF13A (Q9EQW7), KIF14 (L0N7N1), KIF15 (Q6P9L6), KIF16B (B1AVY7), KIF17 (Q99PW8), KIF18A (Q91WD7), KIF18B (Q6PFD6), KIF19 (Q99PT9), KIF20A (P97329), KIF20B (Q80WE4), KIF21A (Q9QXL2), KIF21B (Q9QXL1), KIF22 (Q3V300), KIF23 (E9Q5G3), KIF24 (Q6NWW5), KIF26A (Q52KG5), KIF26B (Q7TNC6), KIF27 (Q7M6X4), KIF28 (D3YXS5), KIFC1 (Q9QWT9), KIFC2 9 (O08672), and KIFC3 (O35231). Sequences not available from UniProtKB were obtained from NCBI (Sayers et al., 2021): KIF6 (NP_796026.2), KIF13B (NP_001074646), and KIF16A (STARD9, NP_00135786). Alignments were performed using Clustal Omega v.1.2.4 (Madeira et al., 2022) and visualized so that colors indicate percent identity using Jalview (Waterhouse et al., 2009).

**1.3.2 Confocal Microscopy**

*Kif3a^-/-^Kif3b^-/-^* 3T3 cells were seeded on coverslips in 12-well plates at a density of 1x10^5^ cells/well. Approximately 8 hours later, they were transfected with unlabeled wildtype (WT) KIF3A and WT or E250Q KIF3B-mNeonGreen (mNG). Forty-eight hours after transfection, cells were fixed with PHEMO fixative (3.7% paraformaldehyde, 0.05% glutaraldehyde and 0.5% Triton X-100 in PHEMO buffer) for 10 minutes and then briefly rinsed in PHEMO buffer (68mM PIPES pH 6.8, 25 mM HEPES-KOH pH 7.4, 15 mM EGTA pH 8.0, 3 mM MgCl_2_, 10% DMSO; final pH 6.8) without additional permeabilization (Mabit et al., 2002). Microtubules were immunostained with a rabbit anti-β-tubulin (Abcam Cat# ab179513; RRID:AB_3073861; 1:1000) primary antibody and goat anti-rabbit-AlexaFluor 647 (Thermo Fisher Scientific Cat# A-21244, RRID:AB_2535812; 1:500). Nuclei were visualized with DAPI (Biotium Cat# 40043; 1:10,000). Images were acquired on an inverted confocal microscope (Leica SP8) using a 63x, 1.4 NA oil immersion objective.

**1.3.3 Western Blot**

*Kif3a^-/-^Kif3b^-/-^* 3T3 cells were seeded in 12-well plates at a density of 0.7x10^5^ cells/well. Six hours later, they were transfected with soluble mNG and mCh or wildtype (WT) *Mm*KIF3A-mCh and either WT, E250Q, L523P, or A334T *Mm*KIF3B-mNG. Thirty-six hours after transfection, crude lysates were collected as previously described (Engelke et al., 2016). Lysates were diluted in lysis buffer containing no Triton X-100 and protein concentration determined via Bradford assay (Pierce Cat# 23200). Twenty-five µg of total protein from each sample was boiled at 95°C for 5 minutes in 1x Laemmli buffer (Biorad Cat# 1610747) with 200 mM DTT (Sigma Cat# 43815) and loaded into a 7.5% TGX FastCast Acrylamide gel (Biorad Cat# 1610170) along with EZ-Run Prestained Rec Protein ladder (Fisher BioReagents Cat# BP36031 ). Following electrophoresis, proteins were transferred to a PVDF membrane (Invitrogen Cat# IB401002) using the Invitrogen iBlot gel transfer system. The membrane was blocked in 5% nonfat milk in TBS-T and then incubated overnight at 4°C with the primary antibodies rabbit anti-KIF3B (Cell Signaling Technologies Cat# 13817; RRID:AB_2798321; 1:1000) and rabbit anti-β-tubulin (Abcam Cat# ab179513; RRID:AB_3073861; 1:200,000). Following three washes in block, the membrane was incubated at room temperature for 1 hour with the secondary antibody goat anti-rabbit-horseradish peroxidase (Jackson Immunoresearch Cat# 111-035-144; RRID:AB_2307391; 1:10,000). Following two washes in block and two washes in PBS, the signal was developed with Clarity Western ECL Substrate (Biorad Cat# 1705060S) and the membrane was exposed to autoradiography film (Lab Scientific Cat# XAR ALF 2025). Subsequently, developed film was scanned into the computer and bands were analyzed with Fiji ImageJ Gel Analyzer (Schindelin et al., 2012).

**References**

Engelke, M. F., Winding, M., Yue, Y., Shastry, S., Teloni, F., Reddy, S., et al. (2016). Engineered kinesin motor proteins amenable to small-molecule inhibition. *Nat. Commun.* 7, 11159. doi: 10.1038/ncomms11159

Mabit, H., Nakano, M. Y., Prank, U., Saam, B., Döhner, K., Sodeik, B., et al. (2002). Intact Microtubules Support Adenovirus and Herpes Simplex Virus Infections. *J. Virol.* 76, 9962–9971. doi: 10.1128/jvi.76.19.9962-9971.2002

Madeira, F., Pearce, M., Tivey, A. R. N., Basutkar, P., Lee, J., Edbali, O., et al. (2022). Search and sequence analysis tools services from EMBL-EBI in 2022. *Nucleic Acids Res.* 50, W276–W279. doi: 10.1093/nar/gkac240

Sayers, E. W., Bolton, E. E., Brister, J. R., Canese, K., Chan, J., Comeau, D. C., et al. (2021). Database resources of the National Center for Biotechnology Information. *Nucleic Acids Res.* 50, D20–D26. doi: 10.1093/nar/gkab1112

Schindelin, J., Arganda-Carreras, I., Frise, E., Kaynig, V., Longair, M., Pietzsch, T., et al. (2012). Fiji: an open-source platform for biological-image analysis. *Nat. Methods* 9, 676–682. doi: 10.1038/nmeth.2019

The UniProt Consortium (2023). UniProt: the Universal Protein Knowledgebase in 2023. *Nucleic Acids Res.* 51, D523–D531. doi: 10.1093/nar/gkac1052

Waterhouse, A. M., Procter, J. B., Martin, D. M. A., Clamp, M., and Barton, G. J. (2009). Jalview Version 2--a multiple sequence alignment editor and analysis workbench. *Bioinforma. Oxf. Engl.* 25, 1189–1191. doi: 10.1093/bioinformatics/btp033
